# Production of arabitol from glycerol by immobilized cells of *Wickerhamomyces anomalus* WC 1501

**DOI:** 10.3389/fbioe.2024.1375937

**Published:** 2024-04-10

**Authors:** Raffaella Ranieri, Francesco Candeliere, Laura Sola, Alan Leonardi, Maddalena Rossi, Alberto Amaretti, Stefano Raimondi

**Affiliations:** ^1^ Department of Life Sciences, University of Modena and Reggio Emilia, Modena, Italy; ^2^ Biogest-Siteia, University of Modena and Reggio Emilia, Reggio Emilia, Italy

**Keywords:** glycerol, arabitol, immobilized cells, *Wickerhamomyces anomalus*, biorefinery, airlift

## Abstract

Polyalcohols such as arabitol are among the main targets of biorefineries aiming to upcycle wastes and cheap substrates. In previous works *Wickerhamomyces anomalus* WC 1501 emerged as an excellent arabitol producer utilizing glycerol. Arabitol production by this strain is not growth associated, therefore, in this study, pre-grown cells were entrapped in calcium alginate beads (AB) and utilized for glycerol transformation to arabitol. Flasks experiments aimed to assess the medium composition (i.e., the concentration of inorganic and organic nitrogen sources and phosphates) and to establish the appropriate carrier-to-medium proportion. In flasks, under the best conditions of ammonium limitation and the carrier:medium ratio of 1:3 (w/v), 82.7 g/L glycerol were consumed in 168 h, yielding 31.2 g/L arabitol, with a conversion of 38% and volumetric productivity of 186 mg/mL/h. The process with immobilized cells was transferred to laboratory scale bioreactors with different configurations: stirred tank (STR), packed bed (PBR), fluidized bed (FBR), and airlift (ALR) bioreactors. The STR experienced oxygen limitation due to the need to maintain low stirring to preserve AB integrity and performed worse than flasks. Limitations in diffusion and mass transfer of oxygen and/or nutrients characterized also the PBR and the FBR and were partially relieved only in ALR, where 89.4 g/L glycerol were consumed in 168 h, yielding 38.1 g/L arabitol, with a conversion of 42% and volumetric productivity of 227 mg/mL/h. When the ALR was supplied with successive pulses of concentrated glycerol to replenish the glycerol as it was being consumed, 117 g/L arabitol were generated in 500 h, consuming a total of 285 g/L glycerol, with a 41% and 234 mg/L/h. The study strongly supports the potential of *W. anomalus* WC 1501 for efficient glycerol-to-arabitol conversion using immobilized cells. While the yeast shows promise by remaining viable and active for extended periods, further optimization is required, especially regarding mixing and oxygenation. Improving the stability of the immobilization process is also crucial for reusing pre-grown cells in multiple cycles, reducing dead times, biomass production costs, and enhancing the economic feasibility of the process.

## 1 Introduction

The transition towards a more sustainable industrial ecosystem hinges on the effective utilization of second-generation feedstock. This strategic approach plays a crucial role in achieving the goals of a circular economy by preventing the accumulation of side streams as waste and concurrently reducing reliance on fossil fuels. Within this framework, crude glycerol, a byproduct from biodiesel or soap industries, emerges as a renewable feedstock with a rapidly increasing availability, mirroring the growth of the biodiesel market (Yang et al., 2012). Undesirably, its substantial impurity content limits its direct industrial applications (Johnson and Taconi, 2007). However, one promising avenue involves employing crude glycerol as a carbon source for microbial fermentative processes in biotechnological industries. Numerous microorganisms, demonstrated to efficiently convert glycerol into value-added chemicals, have been studied and proposed to establish new, profitable biorefineries that align that align seamlessly with the principles of the circular economy with the principles of the circular economy (Crosse et al., 2019).

Polyols such as arabitol and xylitol have gained recognition among these top-tier value-added chemicals, attracting growing scientific interest and establishing themselves as key players in the sustainable industrial landscape (Werpy and Peterson, 2004; Erickson et al., 2012; Raimondi et al., 2023). Arabitol, a five-carbon polyol, have chemical characteristic that make it interesting for industrial uses, such as high sweetening power, low caloric content, and the ability to inhibit the growth of cariogenic bacteria (Amaretti et al., 2020). For these reasons it is as a versatile compound with significant potential applications in the food, oral-healthcare, and pharmaceutical industries. Notably, arabitol emerges as a possible substitute for the well-established xylitol, contributing further to the diversification of sustainable alternatives in various industrial sectors (Yang et al., 2021).

While the industrial manufacture of D-arabitol traditionally involves the chemical reduction of lactones of arabinonic and lyxonic acids, requiring an expensive catalyst, high temperature, and costly separation steps to eliminate by-products (Kordowska-Wiater, 2015), an alternative lies in yeast fermentation from glucose, sucrose, and glycerol. Arabitol, as well as other polyols, represents a potential carbon source entering microbial metabolic pathway or serves physiological functions acting as an osmoregulatory compounds (Jennings, 1984). Many fungal genera (e.g., *Candida*, *Debaryomyces Hansenula*, *Yarrowia*, *Wickerhamomyces*) have been identified as arabitol producers either from sugars or other polyols. Yeast species such as *Debaryomyces hansenii*, *Debaryomyces prosopidis*, *Candida quercitrusa*, and *Hansenula hanomala* have been reported to produce and secrete arabitol from glycerol (Koganti et al., 2011; Koganti and Ju, 2013; Yoshikawa et al., 2014; Filippousi et al., 2022). Nevertheless, the presently reported yields and titers remain below the competitive values required for industrial-scale applications, thus limiting the bioconversion of arabitol from glycerol to laboratory scale. A recent study by Raimondi et al. (2022) demonstrated promising results, reporting high values of yield (Y_P/S_) and arabitol titer using the strain *Wickerhamomyces anomalus* WC 1507 in a fed-batch process with synthetic medium and glycerol as the sole carbon source.

The shift from laboratory-scale to industrial-scale processes in designing biorefineries necessitates the exploration of scalable approaches. Immobilized cell systems stand out as a promising option, offering benefits such as straightforward biomass recovery, reusability in consecutive fermentation batches, and the capability to maintain high cell concentrations without compromising the biocatalyst’s catabolic ability (Moreno-Garcia et al., 2018).

Numerous techniques for immobilizing cells have been explored, including adsorption on a support surface, mechanical containment behind a barrier, self-immobilization, and entrapment in a porous matrix (Jain et al., 2023). Among these approaches, the entrapment in a porous matrix, particularly using calcium alginate, has been extensively investigated due to its cost-effectiveness, straightforward bead preparation process, and mild operating conditions (Clements et al., 2015).

Critical to the scale-up of immobilized cell systems is the implementation of a suitable bioreactor design. An effective bioreactor design must ensure appropriate fluid dynamic conditions and facilitate efficient mass and heat transfer to and from the biocatalyst surface (Obradovic et al., 2004). Immobilized cells systems generally consist of three phases: solid (the carrier or aggregate), liquid (the medium), and gas (air, oxygen, or other gas feeds) (Verbelen et al., 2006). Various reactor designs have been considered for immobilized yeast fermentation. Packed bed reactors offer simplicity and plug flow but face challenges in maintaining ideal conditions due to issues like channeling and mass transfer limitations. Fluidized bed reactors allow intense mixing with reduced carrier abrasion but necessitate careful consideration of density differences to prevent wash-out. Airlift reactors provide vigorous circulation through gas injection but limit the choice of immobilizing particles. Stirred tank reactors increase mass transfer rates with forced agitation but pose concerns regarding support and yeast cell damage (Obradovic et al., 2004; Brányik et al., 2005).

In this study, the strain *W. anomalus* WC 1501 was entrapped in calcium-alginate beads for glycerol-to-arabitol production. The process was initially optimized in terms of medium composition and carrier-medium ratio, and various bioreactor configurations (stirred tank reactor, STR; packed bed reactor, FBR; fluidized bed reactor, FBR; airlift reactor, ALR) were subsequently tested at the laboratory scale to identify the most effective design for the process.

## 2 Materials and methods

### 2.1 Strain, medium and culture condition

All the chemicals were purchased from Sigma-Aldrich (Steinheim, Germany) unless otherwise stated.

The yeast strain *W. anomalus* WC 1501, belonging the collection of the Laboratory of Microbial Biotechnologies (Department of Life Sciences, University of Modena and Reggio Emilia) and already known for its ability to produce arabitol from glycerol (Raimondi et al., 2022), was routinely cultured in MY medium [20 g/L glycerol, 3 g/L yeast extract, 2 g/L (NH_4_)_2_SO_4_, 3 g/L KH_2_PO_4_, 1 g/L K_2_HPO_4_, and 1 g/L MgSO_4_ · 7H_2_O], where the yeast was incubated aerobically at 30°C for 24 h. Unless otherwise stated, arabitol production was carried out in MY medium containing 120 g/L glycerol, with any modifications necessary for specific experiments being explicitly indicated.

The yeast biomass for the flasks experiments with immobilized cells was collected from flask cultures, inoculated 5% (v/v) and grown for 24 h in MY medium. To produce the adequate amount of immobilized biomass for bioreactor experiments, fed-batch fermentations of *W. anomalus* WC 1501 were carried out in laboratory-scale autoclavable fermenters (500 mL Mini Bio, Applikon Biotechnology, Delft, Netherlands). Cultures were started batch-wise in 360 mL MY medium, modified with 9 g/L KH_2_PO_4_ and 3 g/L K_2_HPO_4_, inoculated 5% (v/v). After 12 h, the culture was fed 36 mL of a solution containing 400 g/L glycerol, 60 g/L yeast extract, 40 g/L (NH_4_)_2_SO_4_, and 20 g/L MgSO_4_, at the rate of 2.5 mL/h. The culture was maintained at 30°C and aerated with 0.5 v/v/min filter-sterilized air. Automatic addition of 4 M NaOH prevented the pH was from decreasing below 5.0. Cascade-controlled stirring from 1,000 to 1,700 rpm was used to maintain the dissolved oxygen tension (DOT) at 20%. A defoaming mixture (1:1, v/v) of Xiameter 1520 (Dow Corning, Midland, MI, United States) and polypropylene-glycol was automatically added to prevent foaming.

### 2.2 Immobilization in alginate-beads

The cells of *W. anomalus* WC 1501 were harvested from liquid cultures by centrifugation at 10,000 rpm at 4°C for 5 min. The biomass was washed with saline and mixed (1:2 v/v) with 30 g/L sodium alginate. The suspension was pumped with a peristaltic pump and dropped through a syringe needle into a gently stirred 70 g/L CaCl_2_ solution for gelling in alginate-beads (AB). The AB were kept in the calcium solution at room temperature for 30 min, then they were recovered with the help of a Büchner filter, rinsed with saline, and stored in sterile 70 g/L CaCl_2_ solution at 4°C. All the preparation was carried out in aseptic conditions utilizing sterile materials and solutions. According to the initial concentration, alginate 2% (w/v) AB were obtained.

### 2.3 Shake flasks evaluations: effect nitrogen sources, phosphates, and carrier-to-medium ratio

The effect of medium composition on arabitol production by *W. anomalus* WC 1501 cells immobilized in AB was evaluated in MY medium containing 120 g/L glycerol and modified as appropriate to assay the effect of inorganic nitrogen, yeast extract, and phosphates. All these experiments were set-up in 100 mL flasks filled with 20 mL of medium and 2 g of carriers and incubated aerobically in an orbital shaker at 30°C. The absence or the presence of 2.0 g/L (NH_4_)_2_SO_4_ was evaluated in combination with three levels of yeast extract (0, 0.3, and 3.0 g/L). To evaluate the effect of phosphates concentration, the standard medium containing 3 g/L KH_2_PO_4_ and 1 g/L K_2_HPO_4_, was compared with a modified one where the two phosphates were reduced to 1 and 0.33 g/L, respectively. To investigate the best effect of different carrier-to-medium proportion, six w/v ratios (g/mL) were compared: 1:1, 1:1.5, 1:2, 1:3, 1:4, and 1:10. Processes were monitored by sampling 1 mL every 24 h to measure the turbidity of planktonic cells growth, glycerol consumption, and arabitol production.

### 2.4 Arabitol production in bioreactor with immobilized-cells

**FIGURE 1:**
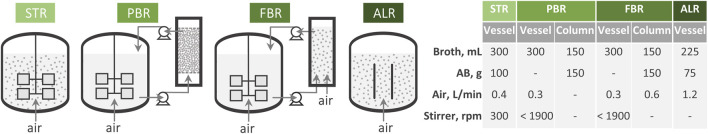
Scheme of the bioreactor configuration and the operational conditions where the AB with immobilized cells of *W. anomalus* WC 1501 were utilized. For each set up, the operational conditions are reported.

AB with immobilized *W. anomalus* WC 1501 cells were utilized in 500 mL Mini Bio bioreactors, modified as appropriate to set up the following configurations: STR, PBR, FBR, ALR (Figure 1). In all the experiments, a carrier-to-medium ratio of 1:3 was utilized. The medium consisted in MY containing 120 g/L glycerol, the reduced concentration of phosphates, and deprived of (NH_4_)_2_SO_4_. For all the configurations temperature of was set at 35°C, the pH was kept at 6.0 by automatic titration with 1 M NaOH, and foaming was prevented by automatic addition of a defoaming mixture (1:1, v/v) of VD 2000 (American Cynamid, Wayne, NJ, United States) and DC 1510 (Dow Corning, Midland, MI, United States). The valued of dissolved oxygen tension (DOT) was continuously measured and registered.

In the STR, 300 mL medium and 100 g of AB were placed in the vessel. The culture was aerated with 0.4 L/min of air, corresponding to 1 v/v/min, and gently mixed at 300 rpm with two 30 mm Rushton turbines.

In the PBR and the FBR, 150 g of AB were confined within a thermostated column with a capacity of 300 mL, connected to a STR apparatus. 450 mL of medium circulated between the column and the STR at the flow of 8 mL/min by means of peristaltic pumps. In the PBR configuration, oxygenation was realized within the STR section by providing 0.3 L/min of air and stirring in the range of 1,000–1,900 rpm to maintain the DOT above 20%. In the FBR configuration, additional 0.6 L/min of air were sparged at the base of the column.

In the ALR, 225 mL medium and 75 g of AB were placed in the vessel where the stirring shaft was removed and a polypropylene draft tube with a diameter of 30 mm was installed to create the riser and downcomer compartments. Mixing and oxygenation were realized by insufflating 1.2 L/min of air (i.e., 4 v/v/min) at the base of the draft tube.

Fermentation runs were conducted in the ALR evaluate the effect of feeding concentrated glycerol. m-MY medium initially containing 120 g/L glycerol was utilized, a carrier-to-medium ratio of 1:3. Whenever consumption decreased below 30 g/L, pulses of 800 g/L glycerol were fed to the system to bring glycerol concentration in the range of 120–130 g/L.

Once the processes started, aliquots of 1 mL were withdrawn daily to measure the turbidity of planktonic cells growth, glycerol consumption, and arabitol production.

### 2.5 Biological and chemical analysis

Turbidity at 600 nm (OD_600_) was utilized to determine the amount of planktonic cells. Glycerol and arabitol concentration (g/L) were analyzed by HPLC apparatus (1200 System, Agilent Technologies, Waldbronn, Germany). Isocratic elution was performed through an ion exclusion column (Aminex HPX-87 H, Bio-Rad, Hercules, CA, United States) with 0.6 mL/min of 5 mM H_2_SO_4_ at 60°C. The sampled aliquots were clarified by centrifugation (10,000 rpm for 5 min) before HPLC measurement (Amaretti et al., 2018).

Yield Y_P/S_ was calculated as gram of arabitol produced (g/L) per gram of arabitol consumed (g/L). Volumetric productivity was calculated by dividing arabitol titer (g/L) by time (hours).

To quantify the free cells in the supernatant or those entrapped in AB, after alginate dissolution, the yeast cells were counted in Thoma’s chamber. To dissolve alginate, 1 g of AB was placed in 9 mL of 10 g/L sodium-citrate solution and vortexed until complete dissolution.

### 2.6 Statistical analysis

All the reported values are means of three separate experiments. t-test and ANOVA followed by Tukey *post hoc* analysis were utilized as appropriate for the comparison of means. Differences were considered statistically significant for *p* < 0.05.

## 3 Results and discussion

The excess of glycerol production by biodiesel manufacturers has transformed it into a waste to be disposed causing an increase in the production costs of biodiesel itself (Carlucci, 2021). The production of added-value chemicals by virtue of environmentally sustainable glycerol conversions, such as the biotechnological ones, can generate additional revenue for the existing biofuel industries and help to resolve the environmental issues associated with waste management, thus making the process more sustainable (Carlucci, 2021; Chilakamarry et al., 2021). Therefore, bioprocesses utilizing glycerol as substrate for the generation of valuable products such as polyalcohols are attracting increasing interest (Diamantopoulou and Papanikolau, 2023). In this context, the efficient glycerol-to-arabitol converting yeast *W. anomalus* WC 1507 was tested in immobilized cells systems.

### 3.1 Effect of medium composition on arabitol production by immobilized *W. anomalus* WC 1501 cells

Arabitol production from glycerol by *W. anomalus* WC 1501 commenced when ammonium got exhausted from a medium containing an excess of glycerol (Amaretti et al., 2020). This behaviour is similar to that of other yeasts, such as *D. hansenii, D. prosopidis*, and *C. quercitrusa*, where arabitol production is decoupled from growth (Koganti and Ju, 2013; Yoshikawa et al., 2014; Filippousi et al., 2022). A recent study indicated that the production of arabitol is triggered by nitrogen limitation also in *Y. lipolytica* (Vastaroucha et al., 2024), but the conflicting evidence, that arabitol production by this species is growth associated and necessitates a supply of ammonium, also exists in the literature (Yang et al., 2021). The non-growth-associated nature of production was exploited in a successful fed-batch process, where *W. anomalus* WC 1501 was first allowed to grow, and then arabitol production was induced with a pulse and feeding of concentrated glycerol (Raimondi et al., 2022). In this study, the feasibility of the process was explored by utilizing pre-produced cells of *W. anomalus* WC 1501 that were immobilized in AB and brought into contact with a medium containing a high concentration of glycerol. In all the experiments herein presented, the load of *W. anomalus* WC 1501 cells within AB lay in the range of 1.7 × 10^10^–2.0 × 10^10^ cells/g.

**FIGURE 2:**
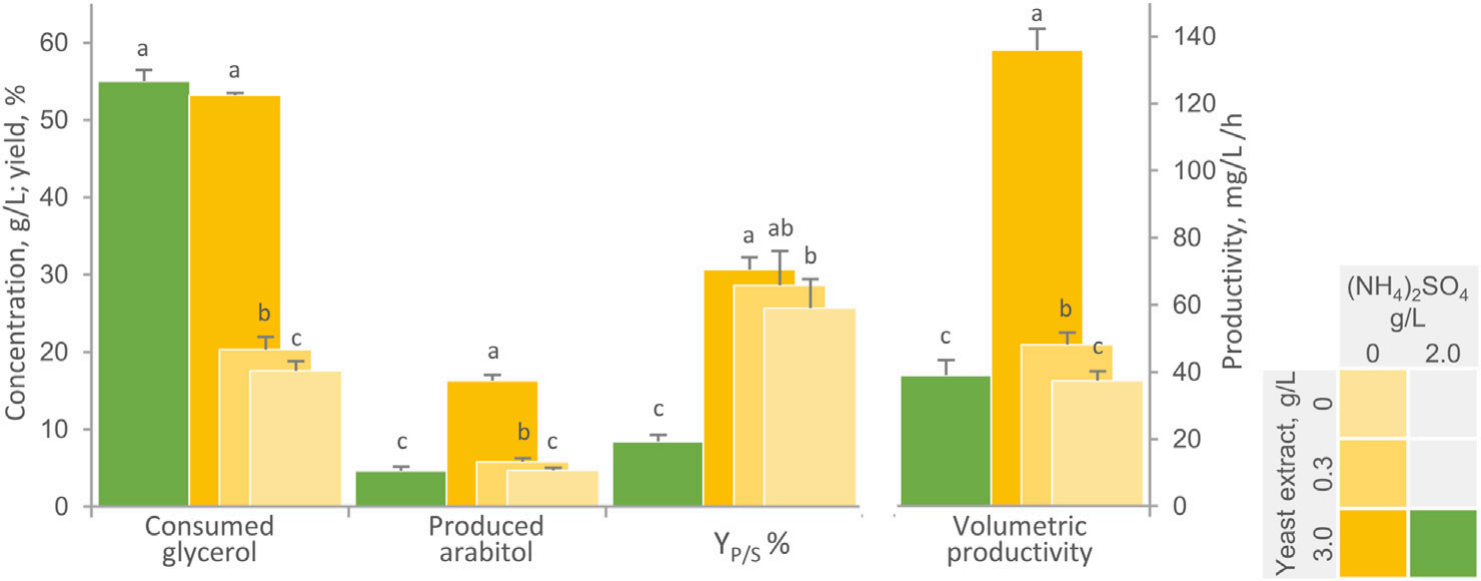
Effect of nitrogen sources on the transformation of glycerol to arabitol by immobilized cells of *W. anomalus* WC 1501 in shake flasks. The values of glycerol consumed, arabitol produced conversion yield, and volumetric productivity after 120 h are reported. Values are means ± SD (*n* = 3). Within each series, means with different letters significantly differ (*p* < 0.05, ANOVA, Tukey *post hoc*).

Since nitrogen limitation is necessary condition also to favor transformation of glycerol into arabitol, the impact of lowered amounts of nitrogen sources on the performance of immobilized cells was evaluated. Shake flasks with a AB:medium ratio of 1:10 were utilized to assess the effect of the absence of inorganic nitrogen in combination with low levels of yeast extract (0 and 0.3 g/L), as opposed to the complete medium containing both 2 g/L NH_4_(SO_4_)_2_ and 3.0 g/L yeast extract (Figure 2). The highest arabitol titre, conversion yield, and volumetric productivity were obtained in the absence of ammonium and in presence of 3 g/L yeast extract (*p* < 0.05). Under these conditions, 53.2 g/L glycerol were consumed in 120 h, yielding 16.3 g/L arabitol with a Y_P/S_ of 30.6% and a productivity of 136 mg/L/h. At the end of the experiment, planktonic cells reached a turbidity (OD_600_) of 15.1. A similar extent of glycerol consumption (*p* > 0.05) was observed in presence of ammonium. However, in these conditions, glycerol utilization likely contributed to the growth of planktonic cells, which reached an OD_600_ of 38.0, while arabitol production was depressed in terms of final titre (4.6 g/L), yield and productivity (*p* < 0.05). This observation confirms the importance of inorganic nitrogen limitation for *W. anomalus* WC 1501 likewise in other yeasts (Nozaki et al., 2003; Filippousi et al., 2019; Filippousi et al., 2022; Raimondi et al., 2022). Nonetheless, the mechanism of such regulation (e.g., a potential role of AMP deaminase as in oleaginous fungi) remains to be clarified in *W. anomalus*.

Nitrogen limitation serves as a pivotal trigger for a range of metabolic events in various yeasts and fungi, including the accumulation of intracellular polyalcohols, polysaccharides, and storage lipids, as well as the release of citric acid (Diamantopoulou and Papanikolau, 2023), thus highly unbalanced cultivation media with high C:N ratios are frequently employed to promote the production of these products, depending on the strain’s capability. For the biosynthesis of polyalcohols in fungi, glycerol must be transformed into dihydroxyacetone phosphate/glyceraldehyde 3-phosphate, which must then enter the pentose phosphate pathway (PPP) through different routes (Carly and Fickers, 2018; Diamantopoulou and Papanikolau, 2023). The pathway leading to arabitol production involves the conversion of the phosphorylated three-carbon molecules into glucose 6-phosphate, which is then processed in the PPP to form ribulose or xylulose, eventually being reduced to arabitol (Koganti et al., 2011; Diamantopoulou and Papanikolau, 2023). The redirection of significant amounts of phosphorylated three-carbon molecules from catabolism and ATP production toward arabitol synthesis likely necessitates conditions where nitrogen becomes limiting for microbial growth.

Reducing the yeast extract concentration to 0.3 g/L or eliminating it had an adverse impact on glycerol consumption by *W. anomalus* WC 1501 (*p* < 0.05) and, as a consequence, also on the amount of arabitol generated, even though the effect on Y_P/S_ was limited. Prior research emphasizes the importance of an appropriate balance of carbon and organic or inorganic nitrogen sources for both growing and resting cells of various yeast species utilized in arabitol production, relying on metabolic activities associated with the pentose cycle (Nozaki et al., 2003; Kumdam et al., 2013). Furthermore, it should not be excluded that yeast extract provided the medium with essential vitamins and/or micronutrients and trace elements that are essential to arabitol production (Kumdam, et al., 2013).

**FIGURE 3:**
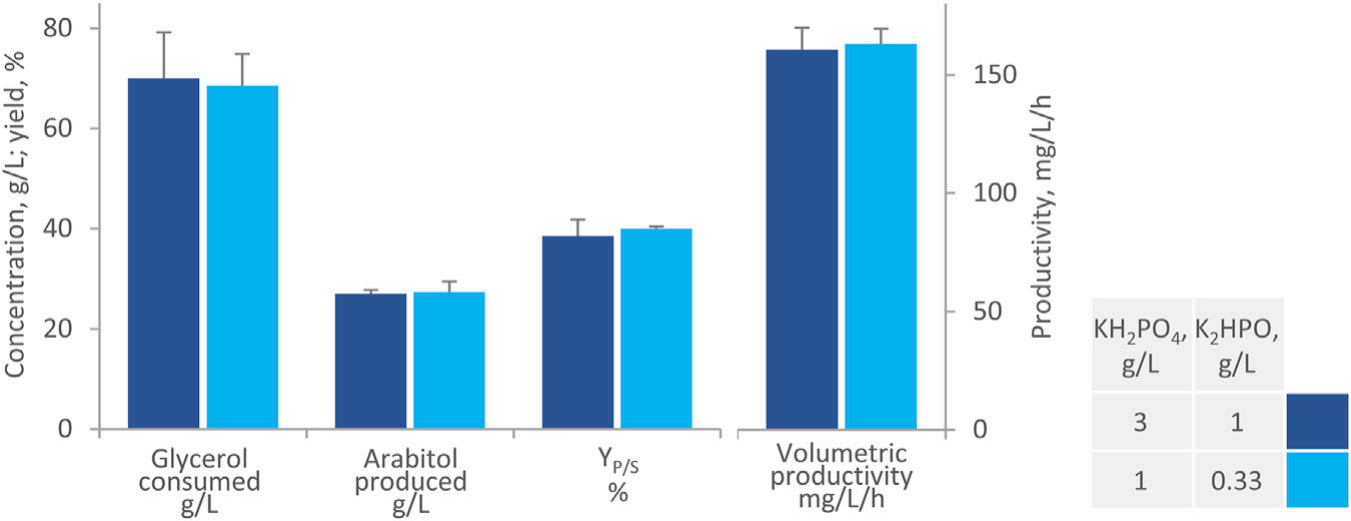
Effect of phosphates on the transformation of glycerol to arabitol by immobilized cells of *W. anomalus* WC 1501 in shake flasks. The values of glycerol consumed, arabitol produced conversion yield, and volumetric productivity after 168 h are reported. Values are means ± SD (*n* = 3). Within each series, * indicates significantly different means (*p* < 0.05, t-test).

Further shake flask experiments focused on reducing the concentration of phosphate salts to one-third, with the aim to improve the stability of AB. The optimal pH for arabitol production is 6, and in bioreactor processes, it is typically maintained through automatic titration. At this pH value, phosphates are prone to form calcium precipitates, sequestering it from alginate and resulting in the gradual dissolution of AB (Pajic-Lijakovic et al., 2015). Flask experiments indicated that reducing phosphates concentration did not significantly affect either glycerol consumption or arabitol production (Figure 3). The medium with the high and the low phosphate concentration yielded similar performance after 168 h (*p* > 0.05), i.e., the consumption of 69.0 g/L g/L glycerol, the production of 27.0 g/L arabitol with a Y_P/S_ of 39% and productivity of 162 mg/L/h. Conversely, concentration of phosphate affected beads integrity at the end of the process: lower values resulted in negligible fragmentation and reduced supernatant turbidity due to calcium precipitates formations and cells release (data not shown). Based on these results, all subsequent tests were conducted with modified MY medium, hereinafter referred to as m-MY, deprived of ammonium sulfate and containing the reduced amount of phosphates.

**FIGURE 4:**
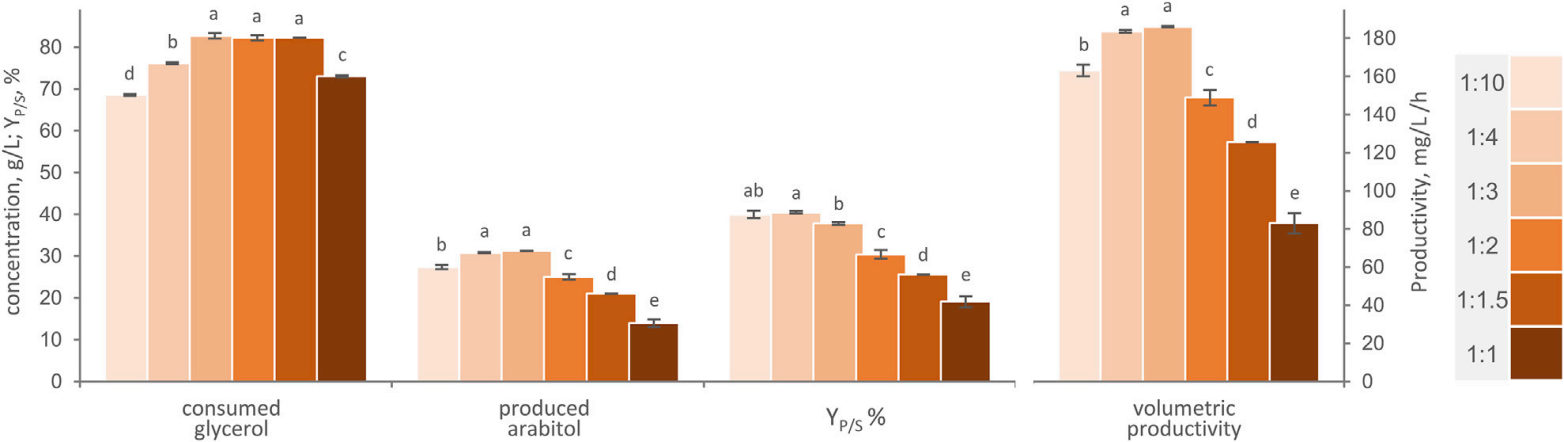
Effect of carrier to-to-medium ratio, in the range between 1:1 and 1:10, on the transformation of glycerol to arabitol by immobilized cells of *W. anomalus* WC 1501 in shake flasks. The values of glycerol consumed, arabitol produced conversion yield, and volumetric productivity after 168 h are reported. Values are means ± SD (*n* = 3). Within each series, means with different letters significantly differ (*p* < 0.05, ANOVA, Tukey *post hoc*).

Six different carrier-to-medium proportions ranging from 1:10 to 1:1 were compared in shake flasks experiments, with the aim to assess the optimal ratio between AB immobilized cells and m-MY (Figure 4). Increasing the amount of AB immobilized cells, from 1:10 to 1:3 carrier-to-medium ratio, led to the best performing conditions in terms of glycerol consumed, arabitol produced, Y_P/S_ and volumetric productivity (*p* < 0.05), clearly due to the increase of catalytic biomass brought in contact with the medium. With a ratio of 1:3, 82.7 g/L glycerol were consumed and yielded 31.3 g/L arabitol in 168 h, with a Y_P/S_ of 37.8% and a volumetric productivity of 186 mg/mL/h. However, a further increase in AB quantity resulted in a progressive reduction in the glycerol consumed and more evidently in the final arabitol titer (*p* < 0.05), with a significant decrease in both conversion yield and volumetric productivity (*p* < 0.05). Despite the greater quantity of catalytic cells in denser biotransformation mixtures, the higher amount of AB in suspension might have impeded mixing, potentially causing limitations in diffusion and mass transfer of oxygen and/or nutrients (Verbelen et al., 2006; Żur et al., 2016).

### 3.2 Effect of bioreactor configuration on processes with immobilized cells

**FIGURE 5:**
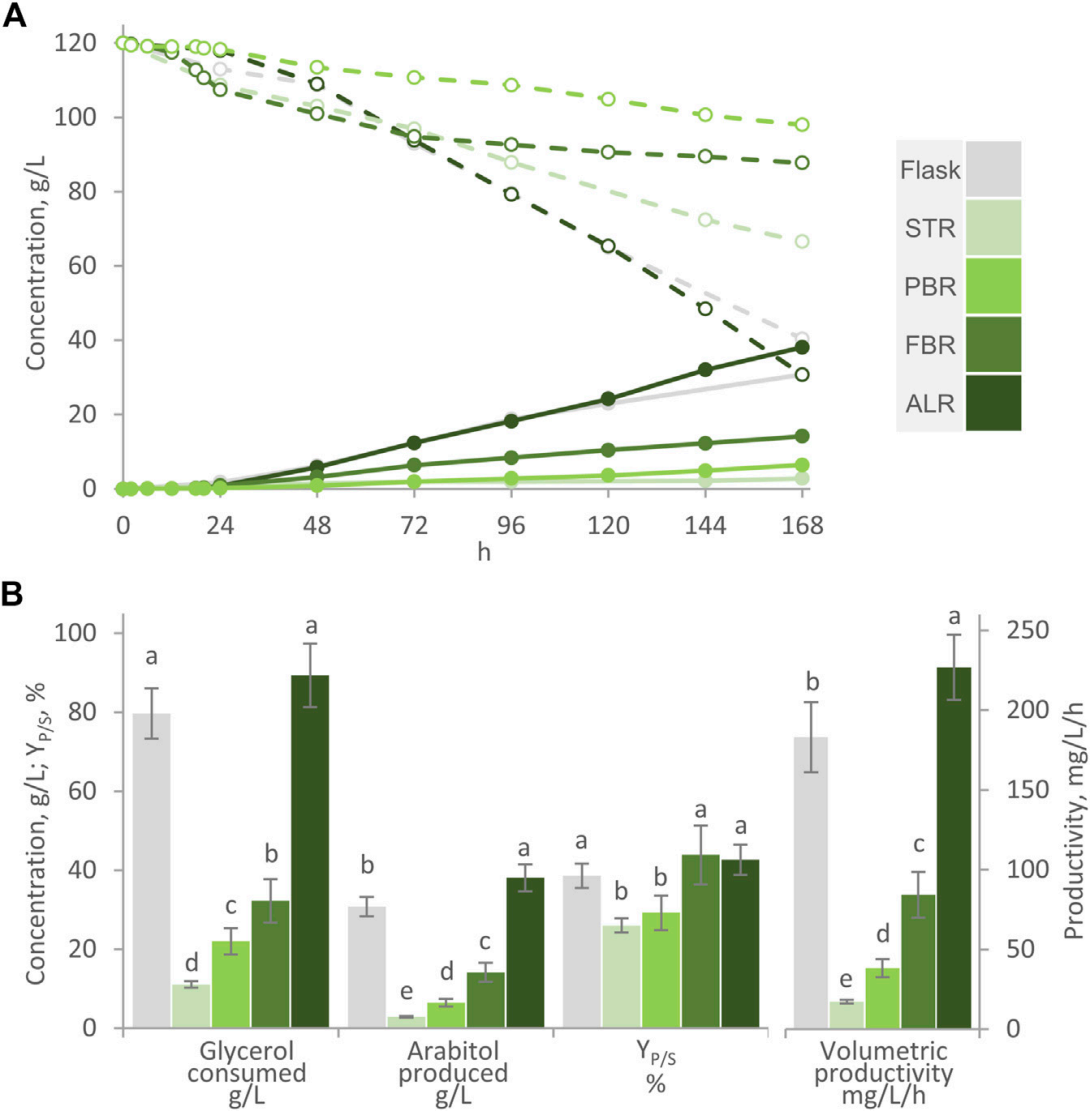
Transformation of glycerol to arabitol by immobilized cells of *W. anomalus* WC 1501 in flasks, Stirred-Tank Reactor (STR), Packed-Bed Reactor (PBR), Fluidized-Bed Reactor (FBR), and Airlift Reactor (ALR). **(A)** Time course of glycerol concentration (dashed lines) and arabitol production (solid lines). The experiments were carried out in triplicate, representative time-courses are reported herein. **(B)** Glycerol consumed, arabitol produced conversion yield, and volumetric productivity after 168 h are reported. Values are means ± SD (*n* = 3). Within each series, means with different letters significantly differ (*p* < 0.05, ANOVA, Tukey *post hoc*).

AB with immobilized cells of *W. anomalus* WC 1507 were utilized in bioreactor experiments with different configurations, i.e., STR, PBR, FBR, and ALR (Figure 1). All the experiments were carried out with a carrier:medium proportion of 1:3, and utilizing 120 g/L glycerol in the modified MY medium, deprived of ammonium sulfate and containing the reduced amount of phosphates. All the processes were carried out with a carrier:medium ratio of 1:3, which provided the systems with 4.2 × 10^9^–4.7 × 10^9^ cells/mL. For all the processes, shake flasks were run in parallel as control (Figure 5).

In the STR process, turbine rotation was limited to 300 rpm to contain the mechanical stress generated by shear forces and prevent the risk of AB disruption. Consequently, it was not possible to prevent oxygen transfer limitation, that occurred since the first hours of process. After 160 h, only 11 g/L glycerol were consumed and 2.9 g/L arabitol were produced, with a Y_P/S_ of 26%. The performance of STR was significantly lower than flask, with regards to the final arabitol titer, the conversion yield, and the productivity (*p* < 0.05). Such failure of the STR configuration could be attributed to oxygen limitation. Previous studies indicated that oxygen demand during arabitol production with this and other yeasts is low and that the level of dissolved oxygen, as long as it remained positive, did not significantly impact arabitol production in fed-batch cultures of *W. anomalus* WX 1507 (Koganti et al., 2011; Filippousi et al., 2022; Raimondi et al., 2022). The inability to increase the oxygen transfer rate in this STR system caused the DOT to drop to 0, thereby hindering the formation of the polyol as observed in other strains (Koganti et al., 2011; Kumdam et al., 2013; Filippousi et al., 2022).

In the PBR, the stirred tank served for the oxygenation of the medium, that continuously circulated between this reservoir and the column where AB were packed. Since AB where absent in the stirred compartment, cascade control of stirring could be applied and effectively prevented the DOT to decrease below 20% through the synergistic action of air insufflation and turbine rotation. After 168 h glycerol consumption and arabitol production were respectively 22.0 and 6.4 g/L with a Y_P/S_ of 29.3% and productivity of 38 mg/L/h, higher than in the STR, but still lower than in the flask (*p* < 0.05). In the FBR, where air was sparged also in the column compartment, the process performance slightly but significantly improved compared to the PBR, consuming 32.2 g/L glycerol and yielding 14.2 g/L arabitol with a Y_P/S_ of 43% and a productivity of 84 g/L/h, but again was inferior compared to the flask (*p* < 0.05). These observations confirmed that mixing and oxygenation is critical and that mass transfer phenomena could have limited the performance of PBR. Probably, the dense packing of alginate beads, coupled with the sole piston-like flow of the culture medium during its ascent through the column, did not meet the nutrients and substrate diffusion requirement for an optimal transformation. Evidently, maintaining the DOT > 20% in the aerated and stirred compartment did not guarantee that oxygen reached all the cells, immobilized in the packed column, with an adequate rate. A transition in the metabolic activity toward a more anaerobic state as result of immobilization was previously established in other yeasts and was reported to negatively affect polyols production (Bisping et al., 1996). This alteration could be attributed to the limited availability of oxygen within the immobilizing material. At the same time, the upward air flow within the column maintained the AB in suspension and likely facilitated the diffusion of glycerol and other nutrients toward the cells and their release of arabitol into the medium.

To further improve mixing and oxygenation of the medium, the bioreactor was reconfigured as an ALR, avoiding the circulation of the medium between physically separated compartments, applying an intense air flow that maintained the AB in suspension and mixed and oxygenated the medium. The performance of the ALR surpassed that of all the other configurations (*p* < 0.05), primarily attributable to the superior oxygenation and mixing of the medium. After 168 h, 89.4 g/L glycerol were consumed in the ALR, to yield 38.1 g/L arabitol, with a Y_P/S_ of 42.6% and a volumetric productivity of 227 mg/L/h.

### 3.3 Efficiency of cells entrapment

**FIGURE 6:**
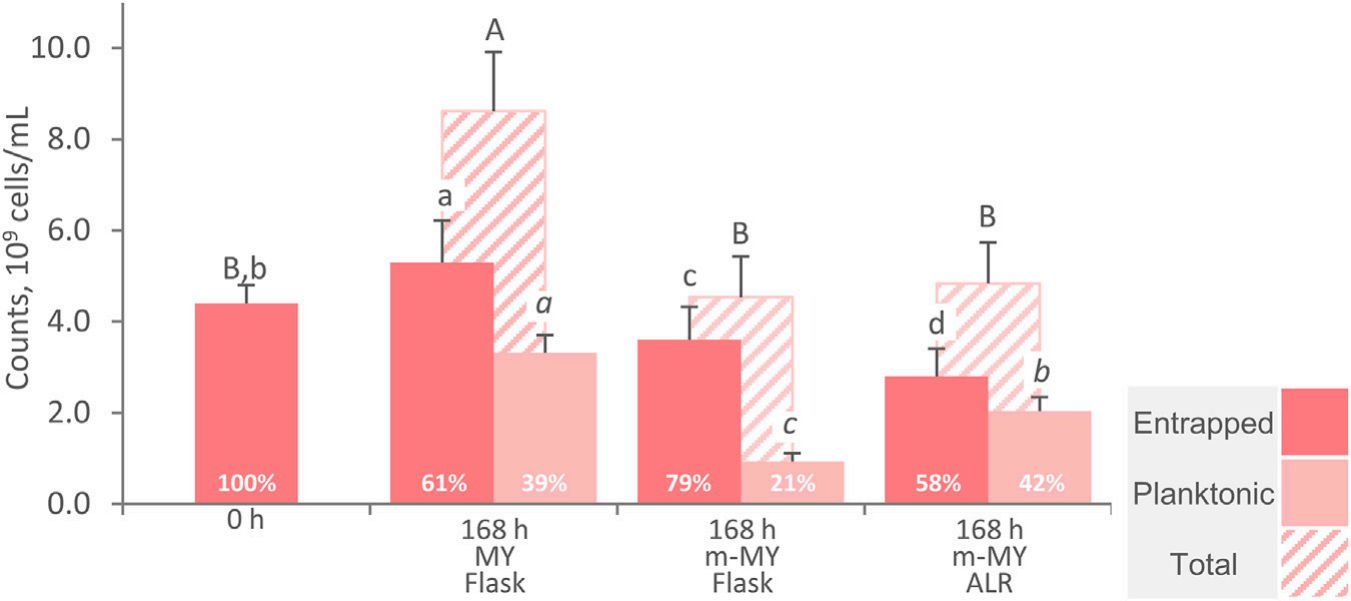
Counts of entrapped, free and total cells of *W. anomalus* WC 1501 at the beginning and after 168 h incubation of AB. MY medium in flasks and m-MY medium in flasks and ALR are compared. Percentages are the proportion of entrapped cells, with respect to the total. Values are means ± SD (*n* = 3). Uppercase, lowercase, and italic letters indicate statistically significant difference between means of total, entrapped, and planktonic cells, respectively (*p* < 0.05, ANOVA, Tukey *post hoc*).

To evaluate the effectiveness of cells entrapment within AB, the cells were counted at the beginning and after 168 h of incubation in flasks with MY and m-MY medium and after 168 h ALR processes in m-MY (Figure 6). All the processes were carried out with a carrier:medium ratio of 1:3, with the system being initially provided with 4.4 × 10^9^ entrapped cells per mL of broth. The complete medium MY, that contained ammonium sulphate, enabled growth up to 8.6 × 10^9^ cells/mL. Such increase of cell counts was characterized by the growth of both cells that remained entrapped within the AB (up to 5.3 × 10^9^ cells/mL, corresponding to the 61% of the total) and those that, once escaping the alginate reticulum, grew in a planktonic state (up to 3.3 × 10^9^ cells/mL). The utilization of m-MY medium, that is depleted in inorganic nitrogen, did not yield any significant change in total cells counts (*p* > 0.05) in both the flask and the ALR processes. Since growth was hindered by nitrogen limitation, the presence of cells in the planktonic condition likely resulted from their gradual release into the medium. This release might have occurred due to the swelling of the carrier and the loosening of the calcium alginate reticulum. The retention of cells within the entrapping carrier was greater in flasks than in ALR processes (*p* < 0.05), with 3.6 × 10^9^ cells/mL being retained in the former and 2.8 × 10^9^ cells/mL in the latter, corresponding to 79% and 58% of the total, respectively. Although the pneumatic bioreactors such as the ARL present no focal point of energy dissipation and shear forces are very homogeneous and mild (Guieysse et al., 2011), it is likely that the improved mixing obtained with this plant configuration was sufficient to determine the partial release of yeasts cells from AB (Pajic-Lijakovic et al., 2015; Lopez-Manchero et al., 2021). Unlike other yeast processes, where the medium permitted growth within particles (Ranieri et al., 2024), the decrease of the cell load associated with AB inevitably reduce the reusability of the carrier in consecutive production runs. Therefore, additional research efforts are necessary to improve the stability of immobilization without hindering the diffusion phenomena, which have already proved to be critical.

### 3.4 Pulsing

**FIGURE 7:**
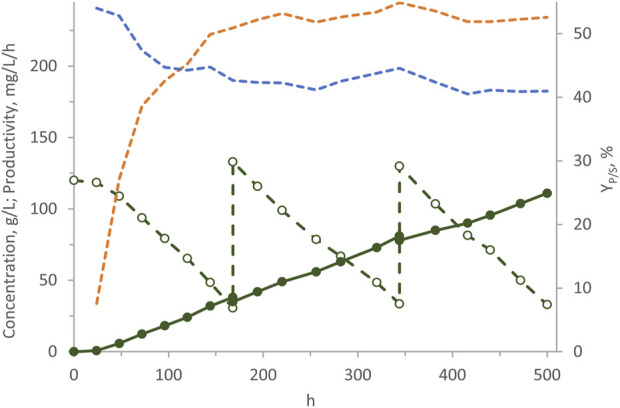
Transformation of glycerol to arabitol by immobilized cells of *W. anomalus* WC 1501 in ALR, subjected to consecutive pulses of concentrated glycerol. Time course of glycerol concentration (dashed green), arabitol production (solid green), Y_P/S_ (dashed blue), and volumetric productivity (dashed orange). The experiment was carried out in triplicate, with a representative time-courses being reported herein.

In a previous study with *W. anomalus* WC 1501, the highest arabitol production was attained through fed-batch processes (Raimondi et al., 2022). This approach consisted in feeding a pre-grown high cell density culture with a concentrated glycerol solution, aiming to maintain a high glycerol concentration in the culture. In the present study, fermentation runs were conducted in the ALR using m-MY medium initially containing 120 g/L glycerol and consecutive pulses of concentrated glycerol were fed to the system with the aim of replenishing glycerol concentration to values within the range of 120–130 g/L whenever consumption reduced it to <30 g/L (Figure 7). In 500 h of process, 117 g/L arabitol were generated, consuming a total of 285 g/L glycerol. Throughout the process Y_P/S_ settled to the value of 41%, while the volumetric productivity progressively increased, tending to plateau of and 234 mg/L/h. Despite these values are not as high as those attained in the most efficient fed-batch suspended process, which yielded 265 g/L arabitol after 325 h, this experiment served as evidence that the cells of *W. anomalus* WC 1501 retained their vitality and activity over an extended period. In fact, glycerol consumption and arabitol production continued linearly throughout the entire process yielding one of the highest arabitol titres ever obtained in an immobilized cells process.

The literature reports several examples of processes utilizing immobilized yeast cells to convert various substrates into polyols, including glycerol, erythritol, and xylitol (Carvalho et al., 2002; (Yewale et al., 2016; Ranieri et al., 2024; Hijosa-Valsero et al., 2022). Additionally, one study describes the production of arabitol from concentrated glucose using immobilized yeast cells (Bisping et al., 1996). To the best of our knowledge, the data presented in this study represent the first description of a process utilizing immobilized yeast cells for arabitol production from glycerol. Compared with submerged yeast cultures using glycerol, the process presented in this study demonstrates superior or comparable performance to the majority of other studies summarized in Table 1, even though it is still less efficient than the optimized fed-batch process for the same strain (Raimondi et al., 2022).

**TABLE 1:** Comparison of yeast processes transforming glycerol into arabitol.

Strain	Glycerol	Mode		Arabitol, g/L	Y_P/S_	Productivity, g/L/h	References
*C. quercitrusa* NBRC 1022	Crude	B	S	85	41	0.35	Yoshikawa et al. (2014)
*D. hansenii* NRRL-Y-7483	Pure	B	S	40	55	0.33	Koganti and Ju (2013)
*D. prosopidis* FMCC Y69	Crude	SF	S	57	48	0.11	Filippousi et al. (2022)
*Y. lipolitica* LMBF Y-46	Pure	B	S	14	9	0.05	Papanikolaou et al. (2020)
*Y. lipolytica* FMCC Y-74	Crude	B	S	10	10	0.03	Vastaroucha et al. (2021)
*Y. lipolytica* NRRL Y-323	Crude	B	S	17	62	0.07	Vastaroucha et al. (2024)
*Y. lipolytica* ARA9	Crude	FB	S	119	49	1.10	Yang et al. (2021)
*W. anomalus* WC1501	Pure	FB	S	265	74	0.82	Raimondi et al. (2022)
*W. anomalus* WC1501	Pure	B	IC	38	42	0.23	This study
*W. anomalus* WC1501	Pure	FB	IC	117	42	0.23	This study

Mode: SF, shake flasks; B, batch in bioreactor; FB fed-batch in bioreactor; S, submerged culture; IC, immobilized cells.

## 4 Conclusion

The yeast *W. anomalus* WC1501 has once again demonstrated as one of the leading arabitol producers from glycerol. The data presented in this study strongly support its potential for application in processes involving immobilized cells for the efficient conversion of glycerol into arabitol. The encouraging potential of this yeast lies in its demonstrated ability to remain viable and active over extended periods and its capability to produce arabitol decoupled from growth. However, despite the promising results herein presented, various aspects of the process demand further optimization, particularly in relation to the critical parameters of mixing and oxygenation. Additionally, the stability of the immobilization process necessitates to be improved, as it holds the potential to facilitate the reuse of pre-grown cells of *W. anomalus* WC1501 across multiple production cycles, thus reducing the dead times and the costs related to biomass production and contributing to the economic feasibility of the process.

## Data Availability

The raw data supporting the conclusion of this article will be made available by the authors, without undue reservation.
